# Exploring Older Adults' Perceptions of Using Digital Health Platforms for Self-Managing Musculoskeletal Health Conditions: Focus Group Study

**DOI:** 10.2196/55693

**Published:** 2024-08-01

**Authors:** Sophie Clohessy, Christian Kempton, Kate Ryan, Peter Grinbergs, Mark T Elliott

**Affiliations:** 1 WMG University of Warwick Coventry United Kingdom; 2 EQL Ltd Lancashire United Kingdom; 3 School of Sport, Exercise and Rehabilitation Sciences University of Birmingham Birmingham United Kingdom

**Keywords:** musculoskeletal, digital health platform, physiotherapy self-management, digital triaging, phone app, qualitative, focus group, mobile phone

## Abstract

**Background:**

Digital technologies can assist and optimize health care processes. This is increasingly the case in the musculoskeletal health domain, where digital platforms can be used to support the self-management of musculoskeletal conditions, as well as access to services. However, given a large proportion of the population with musculoskeletal conditions are older adults (aged ≥60 years), it is important to consider the acceptability of such platforms within this demographic.

**Objective:**

This study aims to explore participants’ opinions and perceptions on the use of digital platforms for supporting the self-management of musculoskeletal conditions within older adult (aged ≥60 years) populations and to gather their opinions on real examples.

**Methods:**

A total of 2 focus groups (focus group 1: 6/15, 40%; focus group 2: 9/15, 60%) were conducted, in which participants answered questions about their thoughts on using digital health platforms to prevent or manage musculoskeletal conditions. Participants were further presented with 2 example scenarios, which were then discussed. Interviews were audio recorded, transcribed, and analyzed thematically. Participants were aged ≥60 years and with or without current musculoskeletal conditions. Prior experience of using smartphone apps or other digital health platforms for musculoskeletal conditions was not required. Focus groups took place virtually using the Teams (Microsoft Corp) platform.

**Results:**

A total of 6 themes were identified across both focus groups: “experiences of digital health platforms,” “preference for human contact,” “barriers to accessing clinical services,” “individual differences and digital literacy,” “trust in technology,” and “features and benefits of digital health technologies.” Each theme is discussed in detail based on the interview responses. The findings revealed that most participants had some existing experience with digital health platforms for preventing or managing musculoskeletal conditions. Overall, there was a lack of trust in and low expectations of quality for digital platforms for musculoskeletal health within this age group. While there was some concern about the use of digital platforms in place of in-person health consultations, several benefits were also identified.

**Conclusions:**

Results highlighted the need for better communication on the benefits of using digital platforms to support the self-management of musculoskeletal conditions, without the platforms replacing the role of the health care professionals. The concerns about which apps are of suitable quality and trustworthiness lead us to recommend raising public awareness around the role of organizations that verify and assess the quality of digital health platforms.

## Introduction

### Background

Musculoskeletal health conditions are highly prevalent, with chronic conditions such as osteoarthritis affecting an estimated 8.5 million people in the United Kingdom. Moreover, more than half of the adults aged ≥75 have sought treatment for osteoarthritis [1]. Similarly, back pain affects 10 million adults in the United Kingdom and is the top cause of years lived with disability [2]. Physiotherapy services primarily deal with most referrals for musculoskeletal conditions. In the United Kingdom, there is an increasing burden of musculoskeletal conditions [3] and as such increasing pressure on general practitioner (GP) and physiotherapy services. This increasing burden on services has created the opportunity to develop digital health platforms (eg, a computer or remote device that can help patients manage their health remotely, such as a mobile phone app) to support patients with their physiotherapy needs. Since the COVID-19 pandemic, many digital platforms are being used in conjunction with in-person physiotherapy appointments [4]. Such digital platforms have been developed for a range of purposes, including the triaging of patients with musculoskeletal conditions (ie, assessment of patients’ severity of symptoms and signposting to appropriate services) as well as the self-management of musculoskeletal conditions (eg, physiotherapy exercises [5]). Digital health platforms for musculoskeletal conditions have also been shown to improve patient outcomes (eg, in terms of reduced pain [6]) and, further, in terms of physiotherapy interventions, allow a more interactive and engaging approach to the traditional instruction leaflet, subsequently leading to better intervention adherence [7].

### This Study

A large proportion of patients requiring physiotherapy who are awaiting an appointment are older adults, with age greatly increasing the likelihood of musculoskeletal conditions [8,9]. However, older adults are traditionally considered less confident with technology [10]. A recent focus group study investigated the experiences of older adults using a wide range of diabetes apps. One of the key outcomes from this study highlighted that usability was a major barrier, with participants giving an average System Usability Score [11] across all apps of just 48, equating to the lowest grade category based on the percentile classifications from a wide range of studies [12]. Similarly, an investigation into the use of digital tools in clinical research suggested that older adults are less likely to use digital technology [13]. Therefore, given the increased availability and use of digital platforms for accessing physiotherapy services and supporting the self-management of musculoskeletal conditions, it is important to consider the accessibility and acceptance of such platforms for older adults. This study uses a qualitative approach to gather older adult participants’ opinions and perspectives on using technology for managing musculoskeletal conditions. The aims of the study were to (1) identify any prior experience or knowledge of digital tools to support self-management strategies for musculoskeletal problems, (2) gather opinions on the transition from seeing a health professional in person to using more technology-based support, and (3) gather and present opinions on real-world scenarios where digital platforms support access to and provide guidance on physiotherapy. The results give insights into current perceptions on the use of these technologies for managing musculoskeletal conditions in the older adult population, and we provide recommendations on how to ensure such technologies are accepted by and trustworthy to this patient group.

## Methods

### Participants

Participants were aged ≥60 years. We recruited participants regardless of whether they did or did not have any current musculoskeletal conditions. Similarly, we did not require participants to have any prior experience of using smartphone apps or other digital health platforms for managing musculoskeletal conditions or accessing services. This allowed us to gain a more generalized, unbiased view of using digital health technology for supporting musculoskeletal health. Participants were, however, encouraged to share their prior experiences of using technology (if appropriate).

### Recruitment

The focus groups formed part of a wider study that included a trial investigating the feasibility of using smartphone sensors to objectively measure physiotherapy-related functional tests [14]. Some participants took part in both this focus group study and the trial.

The research team used a strategy to recruit from a wide range of community organizations and venues. These included community centers; local charity groups; libraries; supported living accommodations; and religious centers, such as churches and mosques. A total of 21 local organizations were contacted using the research team’s professional networks along with the networks of the West Midlands Clinical Research Network [15]. Paper and digital format flyers were distributed in these locations through relevant contacts at each organization or via an in-person visit by a member of the research team.

Participants were able to register their interest by providing their name and contact details (email address or postal address) via (1) a web-based form, (2) email, or (3) a telephone voice message, details of which were provided on the flyer. The participant information leaflet and consent form were then sent to those who registered their interest (via either email or post).

Following their registration of interest, a research team member contacted each individual, offering 1 of 2 potential dates on which the focus groups were to be held. Participants registering interest in the later stages, when participation in the first focus group was not practical, were offered only the second date. Consent to participate was acknowledged via email before the focus group date (see *Procedure* section).

### Ethical Considerations

This study involved human participants and was approved by the University of Warwick’s Biomedical and Scientific Research Ethics Committee (147/20-21). All participants gave informed consent, by signing a consent form after reading information about the study prior. Participants were able to withdraw from the study along with their data at any point up to 5 days after participation. The focus groups were recorded and transcribed using Microsoft Teams built-in tools. Data were pseudonymized such that any identifiable details were removed and participant quotes were identified by their first initial and focus group session. No patients or public were involved in the design, conduct, reporting, or dissemination plans of this research. All participants who attended the focus group received a £20 (US $26) gift voucher for their participation.

### Procedure

A total of 2 focus groups were conducted virtually using Microsoft Teams (Microsoft Corp; on August 9 and 29, 2023). This method was chosen to remove the need for the participants, recruited across a relatively wide geographical region, to travel to a specific location for the focus group.

Before the focus group, participants were sent an invite with a clickable Microsoft Teams meeting link. Participants were also sent instructions on how to join the Microsoft Teams meeting, a study information sheet, and a consent form. Participants were instructed to read the information sheet and complete the consent form ahead of the focus group (if participants could not complete the consent form electronically, consent was accepted via email). Participants were also asked to complete a web-based demographic form. The form gathered demographic information, namely age, gender, ethnicity, and self-disclosed musculoskeletal conditions (optional), and consent to receive a £20 (US $26) voucher for participation and provided a summary of the study findings.

Each focus group lasted a total of 1 hour 15 minutes. Both focus groups were recorded and transcribed using the built-in functionality in Microsoft Teams.

Before asking any questions, the facilitator (SC) gave a brief presentation that contained a broad definition of (1) digital health platforms and (2) musculoskeletal conditions (Multimedia Appendix 1). Participants were then initially asked 3 questions covering their experiences of self-managing musculoskeletal conditions, the use of technology to manage musculoskeletal conditions, and their feelings on switching to using more technology in place of a health professional (Multimedia Appendix 1).

**Figure 1:**
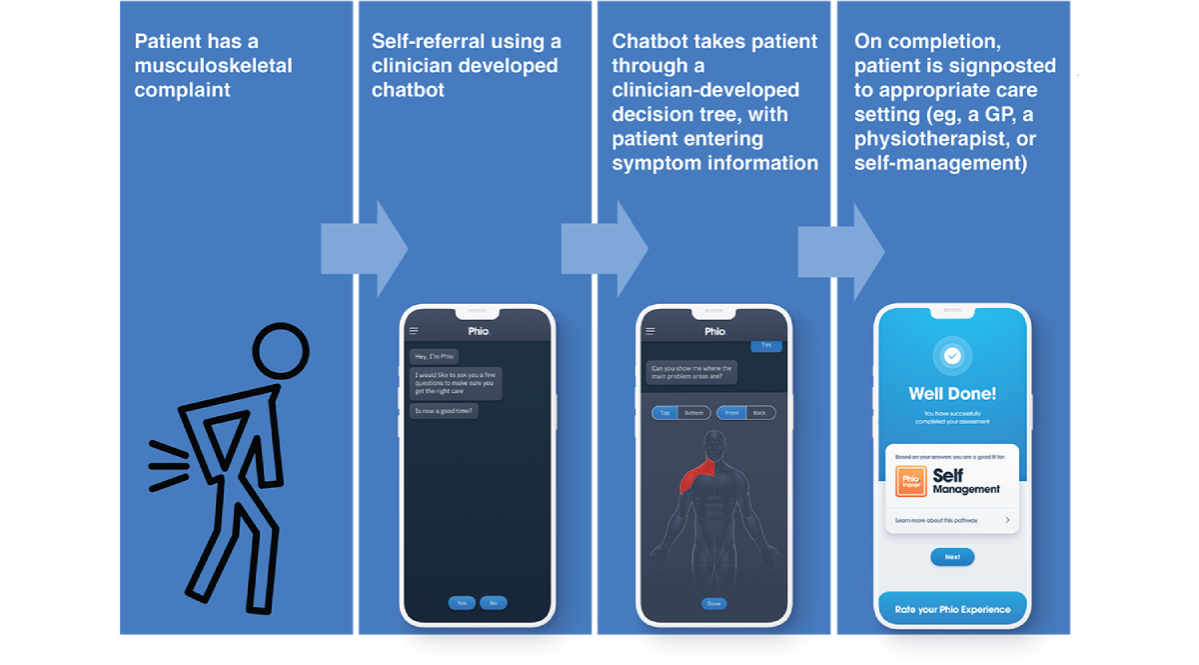
Flowchart describing scenario 1: self-referral using a clinician-developed mobile phone app. GP: general practitioner; NHS: National Health Service.

**Figure 2:**
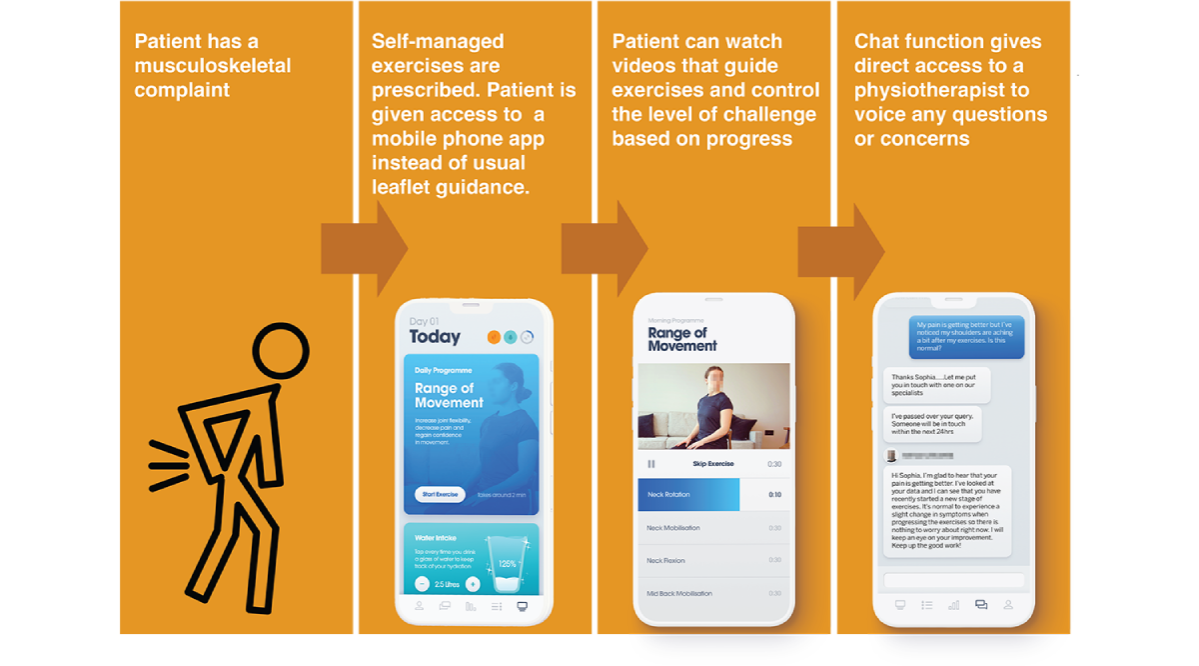
Flowchart describing scenario 2: self-management of physiotherapy exercises using a mobile phone app.

The initial questions were followed by participants being presented with 2 different representative scenarios based on the use of digital health apps to support musculoskeletal conditions. These scenarios were used to provide examples of real apps that were currently used in practice. This provided context to participants who were less familiar with the types of digital health apps currently in use. Scenario 1 related to physiotherapy self-referral using a clinician-developed mobile phone app (Figure 1 [16]). Scenario 2 related to support for the self-management of physiotherapy exercises through the use of a mobile phone app (Figure 2). For each scenario, participants were shown a flowchart describing the app scenario and, on a separate slide, a screenshot of each digital health platform. Alongside the flowchart and image, the facilitator described how each digital health platform worked. Participants were then asked 4 questions about each scenario (Multimedia Appendix 1). The scenarios were kept general; however, they were inspired by apps already developed and used by a UK health technology company (EQL Ltd), which was a partner in the wider project. The images used were from EQL’s apps.

### Data Analysis

Transcripts were anonymized and cross-checked for accuracy by the first author (SC) against the recordings. After each interview, the interviewer, SC, summarized key themes to inform the coding framework. Interview transcripts were then uploaded into the NVivo qualitative analysis software (version 12; QSR International). A thematic analysis was performed using an inductive approach, meaning that codes were derived from the data, and in accordance with the 6-stage model developed by Braun and Clarke [17]. To enhance validity, SC and MTE discussed the resulting coding framework, and themes were identified, reviewed, and defined iteratively. Themes and subthemes are presented and demonstrated by representative quotes.

## Results

### Overview

A total of 15 participants took part in 2 separate focus groups (focus group 1: n=6, 40%; focus group 2: n=9, 60%). During the second focus group, in addition to the 9 participants, a carer acted as a translator for 1 participant. A summary of participant characteristics can be found in Table 1. Of the 15 participants, 12 (80%) reported having current musculoskeletal health conditions; these are listed in Table 2, along with associated *International Statistical Classification of Diseases and Related Health Problems, 10th Revision* codes. A total of 2 (13%) participants had no or did not specifically mention experience of using digital technologies. The remaining participants (n=13, 87%) mentioned they had some experience of using digital technologies relating to musculoskeletal health. In this study, we defined digital technologies relatively broadly and included smartphone apps, smart watches, YouTube (Google LLC), and relevant website content.

**Table 1:** Participant characteristics (N=15).

Participant characteristic			Values
Age (y), mean (SD; range)			71 (8.4; 61-87)
**Gender, n (%)**			
	Woman	10 (67)	
	Man	5 (33)	
	Nonbinary	0 (0)	
**Ethnic group, n (%)**			
	Asian or Asian British	2 (13)	
	Black, African, Caribbean, or Black British	3 (20)	
	White	10 (67)	
**Are there any current musculoskeletal conditions? n (%)**			
	No	3 (20)	
	Yes	12 (80)	

**Table 2:** Health conditions recorded by participants on the demographic form (N=15). Participants could list multiple conditions, so total entries add up to greater than the number of participants.

Reported musculoskeletal conditions	*ICD-10*^a^ mappings	Participants, n (%)
Osteoarthritis	M15.0	4 (27)
Joint pain (hip and knee)	M25.55 and M25.56	2 (13)
Back pain	M54.5 and M54.9	2 (13)
Hypothyroid	E03.9	1 (7)
Peripheral arterial disease	I73.9	1 (7)
Peripheral neuropathy	G62.9	1 (7)
Condition not stated	—^b^	2 (13)
No reported health conditions	—	3 (20)

^a^*ICD-10: International Statistical Classification of Diseases and Related Health Problems, 10th Revision*.

^b^Not applicable.

A total of 6 themes were identified across the transcripts:

- Theme 1: experiences of digital health platforms
- Theme 2: preference for human contact
- Theme 3: barriers to accessing clinical services
- Theme 4: individual differences and digital literacy
- Theme 5: trust in technology
- Theme 6: features and benefits of digital health technologies.

These themes are discussed in detail in the following sections, supported by representative quotes.

### Theme 1: Experiences of Digital Health Platforms

Most participants referenced previous or current use of digital technology to help prevent or manage musculoskeletal conditions. Examples provided included smartwatches, completing physiotherapy exercises or exercise classes via YouTube or Zoom (Zoom Video Communications, Inc). They further referenced walking as an activity they engage in to either prevent or manage musculoskeletal conditions. Some participants referenced the use of digital health platforms (eg, smartwatch) while walking to count their number of steps. It appears specific features on smartwatches encouraged users to engage in more movement (eg, step count and prompt to move when sitting for a certain length of time):

I try to walk 7000 steps a day, I measure on my wrist. I don’t always do it. I used to do 5000, but then I decided that wasn’t enough. So I try to do 7000 if I can.

M, focus group 1

I use my smart watch a hell of a lot. Cause’ it’s an apple one and it has the exercise mode it has the pedometer, it has all that kind of stuff...my watch will tell me when I’m sitting down for too long, so I go and get up and just walk around. So I do utilize the fitness and the health stuff that come with my watch.

L, focus group 2

Apps were highlighted as beneficial when participants encountered barriers (eg, bad weather conditions) that prevented them from walking outdoors:

I like to go for a walk and I’ve got rheumatoid arthritis. So I’m sometimes a bit limited, but I have used apps and to do exercise which were appropriate for me and just, well, keep moving or keep moving anyway. But just to keep moving and you know, just to do my best cause sometimes if the weather’s not good, you don’t feel like going out, do you? So you can do these sort of things indoors, so I found the apps very good.

N, focus group 1

### Theme 2: Preference for Human Contact

Participants expressed concerns when using a digital health platform for an initial musculoskeletal diagnosis or referral. There were concerns that digital health platforms (scenario 1) might fail to identify the full spectrum of a musculoskeletal condition compared to seeing a health professional in person. One of the participants expressed concerns that a more serious diagnosis might be missed if self-diagnosing via information on the internet. There was a sense that an in-person appointment would also cover the wider well-being of a person (eg, identify signals that someone is experiencing domestic abuse):

I mean, if you go online and you start diagnosing yourself...it always ends up to be something else less serious. I couldn’t do that not unless the law changed, but not for me.

S, focus group 2

What you can never get from an app is the face to face, because quite often when people go to the doctor with one thing, the doctor looking at them see’s something else that they hadn’t sometimes realised themselves. In domestic abuse situations, someone can go in regarding something, but the doctor looking at that person can see there’s more to this situation than just what she’s telling me. So you just can’t get that from an app. So this is why I think face to face with always remain a crucial part of the you know, welfare of human being.

J, focus group 2

If I was going to go down the route of asking an app about my symptoms with a chance that it might be self-management, I would have figured out where the self-management things myself first before I would start. If I’ve got to the stage where I’m asking for help, I want help from a person, not from an app.

M, focus group 1

Participants further cited concerns toward carrying out physiotherapy exercises without physical observation or input from an in-person physiotherapist. One of the participants explained how following a hospital appointment, they had been given physiotherapy exercises to complete (the exercises were described on a sheet of paper). They described how they realized they were carrying out the exercises incorrectly only after a family member had observed them:

I was given some exercises to do pelvic floor exercises which I must have been doing for about a month or so and thought I was getting on well. And then my midwife daughter said you’re not doing those right at all. I’d been given a sheet at the hospital to follow, and I thought I was doing exactly what the sheet said. And she said, mum, what you’re doing is useless now. I’ve been doing that for four or five weeks. There are things that you can benefit, but I didn’t know I wasn’t doing the exercises correctly, so I presume thousands of other people in the country who have walked away from hospital with a sheet of paper, get home and think they’re doing them properly and they’re not. But I don’t know how to overcome that because it was only my daughter visiting me that I found out about that.

M, focus group 2

Participants reported further experiences of completing physiotherapy exercises incorrectly without direct observation or supervision. Consequently, some participants appeared to be wary of carrying out physiotherapy exercises alone (scenario 2):

My dad had online physio through his iPad and he was doing this exercise to try and strengthen his glutes. He was doing it and he was turning his whole body and a physio in person would have seen that and known that he wasn’t actually doing the right exercise. So I’m not a fan of online or apps for the consultation and monitoring.

My, focus group 2

I think there is one issue with that, which is how the patient knows that he or she actually does the exercise in a correct way without monitoring by the presence of the physio. I think they need to look at it and then to find some ways in order to ensure that the messages delivered and the exercises are the way the GP wanted or the way their physiotherapist wants.

H, focus group 2

Given these concerns around the lack of person input, a hybrid approach appeared to be favored by participants in which they used digital health platforms alongside some form of face-to-face meetings with a health professional (eg, a general practitioner or physiotherapist). Participants explained a hybrid approach was preferred to ensure they are completing physiotherapy exercises correctly. It was suggested that patients could record themselves completing exercises. These recordings could be sent via the internet to physiotherapists who could track their progress ahead of a face-to-face meeting, rather than as a substitute for a meeting entirely:

I feel I would use it, but I would want a health professional to tell me what to do and then I would go on to technology and then be able to report back to the health professional.

M, focus group 1

And as long as you’ve had the proper consultant consultation with the physio, I don’t see this taking the place of a physio appointment at all in anyway, but in terms of monitoring and feeding back your progress to your physio in advance of the next meeting, because one of the things that I would look for in that like this is to be accountable and to get to the end of the week and say yes I did do that. Yeah, I did that three times a day all. But for the whole week and to have that recorded and for the physio to have access to that.

M, focus group 2

Another participant suggested patients could complete exercises in a group (even virtually, using a platform such as Microsoft Teams), which could help increase their confidence:

So, if you do it with support possibly even with for example a group. Some get together where you like you are now. And you can see us all individually doing the therapies. You can then say oh excuse me you’re doing that incorrectly or something like that. So we get that input in a better way.

L, focus group 2

It was observed that using technology would depend on a patient’s circumstances:

So last time I saw a physiotherapist when I had a blood test from the doctor beforehand to check it wasn’t some biological problem. It was mechanical and then the physiotherapist was a mixture of exercises and also ultrasound treatment. I obviously can’t do that at home and don’t have the skill to apply the ultrasonic probe in the correct manner, so I think it depends on the injury and what the best treatment is, whether you can go a hybrid mode or whether its technology or just physio.

M, focus group 1

### Theme 3: Barriers to Accessing Clinical Services

While expressing a preference for human contact, participants recognized the role of technology when there are barriers to accessing services. Participants referenced seeking help from medical professionals (eg, occupational health practitioners, general practitioners, and physiotherapists) for musculoskeletal conditions and explained how they decided to use technology while waiting to hear back from a health professional. One of the participants described how they had been assigned self-management exercises by their physician but did not receive the exercises. As a result, they sourced their own self-management exercises on the United Kingdom’s National Health Service (NHS) website. A second participant felt it was not always clear which health professional they needed to see (ie, a physiotherapist or a chiropractor) and had experienced a long wait to get a physiotherapy appointment. Due to this long wait, they had engaged in self-management by trying to move their body regularly and using apps:

I had just sciatica not so long ago and it got diagnosed by the doctor. And he said he’d give me some exercises to do, but he never actually did. So when I went on the web and went on the NHS website and found some site, sciatica specific exercises, which I then started doing on a regular basis.

J, focus group 2

I’ve had to wait six months on the NHS to get a physio appointment. I wasn’t sure whether to go to a chiropractor or a physio. And so just thought I’ll just follow the doctor’s advice and wait. And it was a long wait we didn’t go privately and I’m not being seen. I like to go for a walk and I’ve got rheumatoid arthritis so I’m sometimes a bit limited, but I have used apps to do exercises which were appropriate for me and just, well, keep moving.

N, focus group 1

The availability and accessibility of resources were raised by participants. It appears some participants may have access to health professionals more readily. According to one of the participants, patients can access health professionals with greater ease if they pay for their appointments privately. Despite this perceived benefit, another participant noted it was very difficult to obtain a private physiotherapist’s appointment due to long waiting times:

If you’re privileged enough to be able to pay privately, then there will be a few, a lot fewer barriers.

My, focus group 1

I will say at the moment it’s very difficult to see people privately at, whereas at one time you rang and you saw them the next day. I’ve had to wait two months for appointments, even privately.

B, focus group 1

Considering scenario 1 and in light of current waiting lists, a participant saw the benefits of a referral app:

I was just going to say if you’ve sprained knee or have a hand sprain, for example you go to the GP. You’re not going to see the same GP that you’ve seen last time. And if you get referred for Physio, you’ll be lucky to see a physiotherapist for six months. So an app would be more useful because you would have some advice if you like online immediately.

S, focus group 2

In terms of accessing support for self-management via digital means, participants mentioned accessing useful resources on YouTube. One of the participants described how they had found trained physiotherapists on YouTube to follow a program of physiotherapy exercises and stretches:

I found Bob and Brad on YouTube and that really good, really entertaining.

J, focus group 2

A participant described how they already had an experience of scenario 1. However, they noted It was only certain physicians or healthcare organizations that offered the digital services, suggesting not all members of the public can access this service:

It’s already out there. So as far as I’m concerned, I’ve done it. It’s ok, it’s only experimental in certain surgeries and things, but it’s out there.

L, focus group 2

### Theme 4: Individual Differences and Digital Literacy

The topic of inclusivity arose from the focus groups (ie, can individuals aged >60 years successfully access and use digital technology to manage their health?). Participants acknowledged there may be a range of individual differences when it comes to using digital health platforms. There was acknowledgment that users may differ on a variety of characteristics; for example, age and several elements of apps were highlighted as challenging for users. One of the participants expressed concerns toward people in an older age group (aged ≥75 years) using a referral app (scenario 1). They believed this group of people may prefer to speak with a health professional in person. They explained that chatbots can be difficult to use (eg, a chatbot might not recognize certain phrases or words):

I think the older people, older generation, probably people 75-80 don’t feel ready for it. Even just the telephone conversations with doctors. I like to go in person to see my doctor. Whereas when I talk to my children, they think telephone triage is brilliant, so I think it and that’s not to say I mean there’s a lot of very technical people in the older group, but the vast majority, like people have said either or, not, or are distrusting of it. I just think they much on the whole, much prefer seeing someone, even the telephone, does mean you are talking to your doctor, even if you’re not seeing them face to face. Even though you can do video calls with your doctor, I just think that while it’s good I don’t get on with chat bot, I never put the right words in. You’ve got to know what words to use to make the thing effective.

F, focus group 2

Another participant also raised the issue of accessing digital health platforms. It was implied some people may find it more challenging:

I went into hospital and I had to have physio afterwards and that was given as a YouTube thing with the nurse. My only problem with it, I did most of the exercises and things that were required and requested. The only thing I found was I kept losing the YouTube and trying to find the link to it because they sent it as a link. Because you’re not feeling 100%, I didn’t secure it, so I forgot where I put it and so I stopped doing it so probably didn’t complete the course... because at that point I wasn’t feeling physically fit and I wasn’t very well and probably wasn’t thinking the way I would normally think. Apps need to be absolutely simple and as somebody else said, idiot proof.

F, focus group 2

### Theme 5: Trust in Technology

Issues around the legitimacy of some health apps were highlighted. Participants expressed concerns about not knowing whether apps are real or fake:

It’s not beyond the bounds of possibility that you get on to the wrong sort of website and you get the wrong sort of information. You would need to know that what you’ve accessed is in fact a genuine one.

B, focus group 1

Several suggestions were made to help to instill confidence in users. One of the participants suggested that they would feel more comfortable if information or link for a digital health platform came straight from health care professionals rather than an unknown source:

Nowadays we are bombarded with a lot of information. So if there is a direct communication between the people who supply this app and the clinic itself or the hospital, which means that I received the information from the clinic or from the hospital that gives me confidence and that’s giving me trust that I can trust the source of this information. Rather than it is, it is independent from the hospital or from the clinic, which makes me think is this genuine? Is it the same people?

H, focus group 1

There was also the proposal that app developers might wish to include some form of legitimacy mark (eg, kite mark) to help users know that the app has been verified and delivers accurate health care advice:

It needs a sort of kite mark, if you remember what they are, but I don’t know how you do that.

B, focus group 1

Training was also suggested as a method to encourage greater uptake and use of such apps:

I think the issue is not with using them. I think the main issue is we need more training on how to implement, how to use these apps and how to become familiar with them. This is very important if the training is not available then I think it will create some frustration and with the users and users may refuse or may not like to use them.

H, focus group 2

I think until people can be trained to use it a lot better than. You know, it’s people gonna still find it difficult to use. If you feel you’ve pulled a muscle and you’re in pain, you don’t wanna be fiddling about with technology at that time.

L, focus group 1

In the context of scenario 1, a participant suggested that case studies of worked examples would be useful to instill trust in the referral app:

I guess what we really want is case examples, so a worked example where a patient that goes through what the questions are ends up with self-management. Then a different patient, different symptoms ends up with consult your GP. So examples of how it actually works in practise. But what would what they would be looking for to validate it.

M, focus group 1

One of the participants described how they had recently trialed new technology after being invited by their local hospital. They stated that technology needs to become the norm, which will encourage people to try it:

So you know the technology is out there. It’s just you’ve gotta have people who are prepared to try it. And to make it become the norm. Once it’s the norm, then the fear disappears and so on. But at present a lot of this stuff is new, and it’s scary for a lot of people. You take the fear out and then it’s fine.

L, focus group 2

### Theme 6: Features and Benefits of Digital Health Technologies

In the context of scenario 2, participants stated a number of positive views toward the use of videos demonstrating physiotherapy exercises. Using an app to perform physiotherapy exercises was compared to using a piece of paper with static poses on it. One of the main perceived benefits of videos was the ability to stop and start the video according to the individual’s own pace. Participants also compared watching videos to meeting with a medical professional, who may not always have time to demonstrate exercises, or alternatively, appointments are rushed, and patients are unable to clarify what is correct:

Having the videos online...you can see how your body should be positioned, you can see from one position to the next where your body should be. Where sometimes we just get the instructions or guidance on the flat sheet of paper, you’re unsure how to move next or how your body should be positioned so having the video of the graphics or whatever it is online I think would definitely be beneficial for someone who’s going down that route. Also online you can do exercises at your own pace. Whereas sometimes medical professionals are on a clock, so ok do this, do this and then they’re off. Whereas if you’re online, you’ve got time to go how did that one go again? And you can see it again, slow it down, pause it, look again.

J, focus group 2

If you watch an app, it’s easier to understand what you meant to be doing than looking at a sheet cause I find that if you have followed the exercises from you’re trying to, even if you’re lying down, you’re trying to hold the sheet of the air to check that you’re doing the exercise correctly. I found an app quite good really for showing you how to do them.

N, focus group 2

Other benefits of using apps included reducing the need to travel to a physical location for an appointment, saving patients’ time:

I think it is a good idea because it saves time for a patient to travel from his or her home to the clinic or to the hospital.

H, focus group 2

Well, going to the doctor or physician or therapist, it takes more time... the technology is much better for me at my own pace, at my own time.

S, focus group 1

Participants considered a number of benefits of using the chatbot referral app (scenario 1) compared to going directly to a health professional. These benefits included reducing wait times for patients as well as freeing up time for health professionals:

It’s the way forward, ‘cos it means you’re freeing up GP time, you’re freeing up hospital time. So the people who are more needy can actually get that support. You are getting your support is a slightly easier and slightly less cumbersome way I suppose.

L, focus group 2

I was just going to say if you’ve sprained your knee or have a hand sprain, for example you go to the GP. You’re not going to see the same GP that you’ve seen last time. And if you get referred for Physio, you’ll be lucky to see a physiotherapist for six months. So an app would be more useful because you would have some advice if you like online immediately.

S, focus group 2

Participants spoke of adapting digital health platforms to suit their needs. For example, one of the participants described adapting app exercises to suit their own abilities and pain level:

It started off quite gently and that used every part of your body and something that I had to adapt because I’ve also had an operation on my thought and I am a replacement hips, so I’ll just adapt whatever, but yeah. I just think. That’s what you know most of the things you try to do your best, don’t you try to do them and but just adapt them...Yeah, some exercises are found too fast for me and some too slow. I think the pace of exercise is sometimes very helpful. And also whether you can follow it or not.

N, focus group 1

Suggestions were made for future adaptations to apps. There was a desire for information on the timings of each exercise to be specific. Participants expressed a concern that sometimes it is not clear how long they need to hold a certain position for; as a result, they suggested embedding a countdown or timer into the app:

Sometimes it doesn’t say how long you’re to hold a position for.

N, focus group 1

Like how many times should you do an exercise within a minute or like a time scale so that you’re not doing it too quickly or too slowly? You can then try to keep some sort of balance.

N, focus group 1

Another participant described adapting a phone app from its original use of sending medication reminders. The participant described how they instead used the function on the app to remind them to do specific physiotherapy exercises at specific points in the day. This insight suggests that reminders for physiotherapy exercises could be inbuilt into existing physiotherapy apps (scenario 2):

One thing I’ve found quite useful when I was doing my post-surgery exercises was there’s an app...it tells you when to take pills so it reminds you when it’s time to take your medication, but rather than entering medication, I entered each of the exercise and then I’ve got a record of when I done the exercises and it reminded me to do, it reminded me to take the pink tablet, but I’ve actually got that it was leg raises or whatever and I haven’t been able to find something like that, that is for exercise, rather than just adapting the medication one but that was good cause you have records and you could sort of see progress.

My, focus group 1

## Discussion

### Principal Findings

This study aimed to explore older adults’ opinions and perceptions on the use of digital platforms for supporting the self-management of musculoskeletal conditions. Using a focus group approach, we found that most participants had some knowledge or experience of using digital tools for supporting musculoskeletal problems, although this could be something as simple as YouTube guidance videos. As health care organizations are increasingly focusing on the delivery of digital services (eg, the NHS Long Term Plan in the United Kingdom [18]), we further wanted to gather opinions from participants around potentially using technology-based support as an alternative or a complement to traditional face-to-face services. In the initial stages of discussions, participants were skeptical of this and highlighted a lack of trust in the quality and scope of digital services. However, we further included real-world examples of physiotherapy-related digital health technologies into our focus group discussions. By moving away from theoretical perceptions to specific examples, we found participants subsequently recognized the potential advantages of such platforms and how they could provide relevant support to self-managing musculoskeletal health conditions. In the following sections, we discuss the main findings in more detail.

### Experiences of Using Digital Tools for Self-Managing Musculoskeletal Conditions

In this study, we purposefully aimed to recruit participants for the focus group without explicitly requiring them to either have current musculoskeletal conditions or experience of using technology for managing musculoskeletal health. Despite these broad inclusion criteria and recruitment across a diverse set of community groups, we found that most participants (12/15, 80%) had some type of current musculoskeletal condition. Furthermore, most participants had used a form of technology to help self-manage their condition. Around 60% of adults aged >65 years live with a musculoskeletal condition in the United Kingdom [3]. Therefore, it was expected that a significant proportion of our participants would have current musculoskeletal conditions, as observed. It was less expected to find that most of our participants had some experience of using technology to support the management of their musculoskeletal conditions. However, this included the use of exercise and guidance videos on YouTube, a medium that is accessed by 52% of older adults (aged >65 years) in the United Kingdom [19]. A study investigating the quality of physiotherapy videos for older adults on YouTube [20] found that the content was rated as poor or lower when assessed according to DISCERN [21] and Journal of American Medical Association scoring methods. This highlights the need for high-quality digital tools and resources that are easily accessible for older adults to avoid the default option of using nonregulated or low-quality resources.

### Quality and Expectation

There was some dismissiveness around the ability of a digital health platform to diagnose conditions fully or correctly, highlighting a low expectation for the accuracy or quality of a digital service. However, these comments emphasize the disparities between the perspectives of what the technology is trying to do and the scope of what it is actually doing. For example, in scenario 1, the scope of the chatbot tool covers triaging and signposting the most appropriate service. To achieve this, it does not need to diagnose the condition per se (as inferred by a participant) but rather assess the risk and urgency based on the patient’s symptoms and direct to the most relevant service. One of the participants notably raised the need for education on such technologies. Understanding the aim and scope of a platform, along with its limitations, is important. This sets expectations and ensures there is clarity on what functions the platform will and will not perform, particularly in the context of any traditional (eg, human interaction) functions it will replace [22].

A previous study of diabetes apps with older adults highlighted that usability is the main concern [12]. Here, the use of a chatbot raised usability issues, with participants highlighting that chatbots often fail to function correctly unless exactly the correct wording or phrasing is used to describe their condition. This highlights issues with earlier technologies and limitations in health-based chatbots [23]. The recent advent of large language models has created a step change in the interaction with chatbots, creating significantly increased flexibility in the conversational style in terms of inputs and responses [24]. This is likely to make chatbots more practical and, importantly, accessible in the future. For example, chatbots are likely to be able to quickly switch between different languages for conversations [25], without requiring complex remapping of decision trees or separate database content. However, this broader scope of chat ability comes with challenges around maintaining boundaries within which conversations must remain (ie, forcing the chatbot to discuss content outside the scope of the health conditions it is designed for) [24].

### Trust

Participants raised several issues around trusting the use of technology for self-managing their health. Notably, this was in response to the initial questions before specific scenarios were introduced and highlights a general distrust around health technologies and digital tools for health, with a concern that this is reducing the quality of the health care patients receive. Once the specific scenarios were presented (which contained details about the functionality of digital health tools), then positives were identified. An example of this was when scenario 2 demonstrated the use of videos to provide guidance on exercises, rather than the leaflets with photographs or diagrams that participants would normally receive. Participants immediately recognized the advantages of being able to watch and pause the video to gain more detailed guidance. It has further been shown that people can accurately follow the movements of an avatar [26] or video-based movements for physiotherapy [27], confirming the advantages of animated or video-based guidance. However, additional annotation of videos maybe required, with guidance on the timing of an exercise requested for specific details on how long a position should be held, for example. The advantages of using video-based guidance through an app also included being able to do exercises at one’s own pace and being able to repeat and retry exercises without the pressure of a physiotherapist observing. The patient-therapist relationship can be complex, with the physiotherapist often seen as an educator [28]. These comments from participants demonstrate a flip side to in-person consultations, where a health professional can create anxiety rather than reassurance in some cases, possibly due to an expectation that the patient must perform the exercises correctly while under observation.

Issues in trust also arose around how to determine whether a digital health platform is genuine. One of the participants referred to using a scheme similar to a “kitemark,” referring to the British Standard Institution Kitemark symbol used on products to show it is approved by the British Standard Institution [29]. In fact, in the United Kingdom, the Organisation for the Review of Care and Health Apps (ORCHA [30]) assesses and provides quality assurance to digital health and care platforms. At a more regulatory level, the evidence standards framework for digital health technologies [31] has been developed by the UK National Institute for Health and Care Excellence to provide standardized guidance on the levels of evidence needed for the clinical and economic evaluation of digital health technologies. However, these are aimed more toward delivering guidance to developers and commissioners. It could be argued that a more consumer (ie, patient)-focused communication campaign could raise the awareness of ORCHA and hence allow potential users of the apps to quickly check whether they have been reviewed and rated.

### Balancing Face-to-Face Consultations With Technology

It was clear that during the initial questions, participants were highly reluctant to reduce or replace face-to-face time with a health professional for a digital health platform. Participants highlighted that one of the advantages of in-person consultations was around the more comprehensive and wide-ranging assessments, with participants mentioning that clinicians are more likely to diagnose more complex issues or other unrelated health conditions during a consultation for a musculoskeletal health issue. This further extended to a patient safety aspect, with the opportunity for health care professionals to spot cases of domestic abuse during face-to-face consultations. While in-person consultations were clearly important to participants, it was acknowledged that waiting times to see a physiotherapist and other health professionals are continuing to increase [32], and it was highlighted by one of the participants that this is also increasingly the case with accessing private practitioners as well as NHS in the United Kingdom.

Given this, the advantages of a hybrid approach were recognized, keeping in-person consultations but using digital platforms in-between appointments. In reality, this is the most likely use case in the future, but more evidence is required on how this can be optimized [4]. Digital platforms can deliver a way to provide continuous guidance and support during the long periods between in-person consultations [33] and potentially increase adherence to the physiotherapy program [7]. In some cases, apps have built-in communication functions to allow chat or video call with a physiotherapist, further bridging the gap between in-person sessions (eg, [34]). This approach can help reduce the demand for in-person consultations and thus offer the opportunity for optimally timed regular face-to-face sessions complementing self-management using digital platforms [33].

### Strengths and Limitations of This Study

This study has gathered opinions from a diverse sample of older adults. Although ethnicity was not formally recorded, we recruited participants through a mixture of supported living complexes. We purposely aimed for broad inclusion criteria, where participants were not required to have a current musculoskeletal condition or an experience of using technologies to manage musculoskeletal conditions to participate. While this aimed to get a broad range of opinions, we found most participants did have current musculoskeletal conditions and experience of using technologies. Therefore, a more targeted recruitment approach may have helped balance the sample. A limitation of this study is that it may have attracted participants who already used digital health platforms or had an interest in digital health technology. In particular, our requirement to access the focus group via a web-based platform could have excluded certain individuals with less technical literacy. In contrast, however, a web-based platform can make access easier for some groups (eg, those with mobility problems) [13].

Future research could further explore opinions of health professionals toward these digital platforms, thus providing perspectives from both the patient and health professional sides.

### Conclusions

In conclusion, it is promising that digital health platforms for musculoskeletal conditions appear to be used and normalized within this age group. However, to enhance trust in these technologies, there needs to be clear communication around how digital platforms can support and assist with the self-management of musculoskeletal conditions and that their role is to complement rather than reduce or replace the role of the health care professional. In addition, we recommend raising public awareness around the role of organizations (such as ORCHA) that verify and assess the quality of digital health platforms to further enhance trust in the use of these technologies.

## Appendix

Multimedia Appendix 1 Focus group materials.

## Abbreviations

GP: general practitioner
NHS: National Health Service
ORCHA: Organisation for the Review of Care and Health Apps
