# Supplementary material for: Exploring Older Adults' Perceptions of Using Digital Health Platforms for Self-Managing Musculoskeletal Health Conditions: Focus Group Study
Source: JMIR Aging. 2024 Aug 1;7:e55693. doi: 10.2196/55693 (PMC11327635; doi:10.2196/55693)
Supplement: Multimedia Appendix 1 [file aging_v7i1e55693_app1.docx]

**Multimedia Appendix 1 (Focus Group Materials)**

1. **Digital Health Platform Definition**

What is a Digital Health platform?

- A computer or remote device that can help you manage your health remotely. Examples include;
- Smartphone App (e.g., providing guidance, recording progress)
- A Website/YouTube
- Online/remote chat with physiotherapist via Online platform such as Microsoft Teams.
- Digital Health platforms enable you to not have to visit a location and can be conducted anytime anywhere.

1. **Musculoskeletal Health Conditions Definition**

- Musculoskeletal (MSK) conditions affect many people and can affect your joints, bones and muscles and sometimes associated tissues such as your nerves. They can range from minor injuries to long-term conditions.
- Musculoskeletal health conditions can range from common aches and pains, mobility through to post-surgery physio.

1. **Focus Group General Questions**

**Question 1:** **How do you self-manage any conditions?** (e.g., mobility, aches/pains, osteoarthritis, back pain).

*Prompts:* Where do you go to find information? (Physio appointments, yoga etc., in person vs online), Have you experienced any challenges in terms of getting access to treatment? For example, a physio (if required)? Do you seek treatment privately or via NHS? (and possible reasons for choice), If no issues, ask about prevention, what do they do to keep active and use of any apps etc. to support that.

**Question 2: Is anyone a user of technology or has experience of using technology to manage musculoskeletal conditions?** (e.g., mobility, aches/pains, osteoarthritis, back pain).

*Prompts:* Examples of effective platforms and ineffective platforms? When using an app/digital platform, what makes it challenging for you? If haven't used technology, find out why? E.g. tech literacy, wasn't aware, only offered traditional approaches.

**Question 3: How do/would you feel about switching from seeing a health professional in person for advice/treatment to using more technology?**

*Prompts:* Swapping out time with a clinician, more time with technology, do you trust that? Are you happy with switching more to technology? Balance between technology? Concerns?

1. **Questions for each scenario**

**Scenario 1**

What would be your initial thoughts on using this compared to visiting a GP? (prompts- is this something you would use? If not why not?)

Do you perceive there to be any benefits/drawbacks of the App/chatbot?

Would you trust this information?

Do you anticipate anything that would make it challenging for you to use the App/chatbot?

**Scenario 2**

What would be your initial thoughts on using a smart phone app to carry out physiotherapy exercises compared to visiting a physio (in person) more regularly? (prompts- is this something you would use? If not why not?)

Do you perceive there to be any benefits/drawbacks of the smart phone app?

Would you trust this information?

Do you anticipate anything that would make it challenging for you to use the smart phone app?
